# Adjustment of directly measured adipose tissue volume in infants

**DOI:** 10.1038/ijo.2014.48

**Published:** 2014-04-29

**Authors:** C Gale, S Santhakumaran, J C K Wells, N Modi

**Affiliations:** 1 Section of Neonatal Medicine, Chelsea and Westminster Hospital Campus, Imperial College London, London, UK; 2 Childhood Nutrition Research Centre, UCL Institute of Child Health, London, UK

**Keywords:** Body composition, Infants, Magnetic resonance, Adipose tissue, Anthropometry, Body weights and measures

## Abstract

**Background:**

Direct measurement of adipose tissue (AT) using magnetic resonance imaging is increasingly used to characterise infant body composition. Optimal techniques for adjusting direct measures of infant AT remain to be determined.

**Objectives:**

To explore the relationships between body size and direct measures of total and regional AT, the relationship between AT depots representing the metabolic load of adiposity and to determine optimal methods of adjusting adiposity in early life.

**Design:**

Analysis of regional AT volume (ATV) measured using magnetic resonance imaging in longitudinal and cross-sectional studies.

**Subjects:**

Healthy term infants; 244 in the first month (1–31 days), 72 in early infancy (42–91 days).

**Methods:**

The statistical validity of commonly used indices adjusting adiposity for body size was examined. Valid indices, defined as mathematical independence of the index from its denominator, to adjust ATV for body size and metabolic load of adiposity were determined using log-log regression analysis.

**Results:**

Indices commonly used to adjust ATV are significantly correlated with body size. Most regional AT depots are optimally adjusted using the index ATV/(height)^3^ in the first month and ATV/(height)^2^ in early infancy. Using these indices, height accounts for<2% of the variation in the index for almost all AT depots. Internal abdominal (IA) ATV was optimally adjusted for subcutaneous abdominal (SCA) ATV by calculating IA/SCA^0.6^.

**Conclusions:**

Statistically optimal indices for adjusting directly measured ATV for body size are ATV/height^3^ in the neonatal period and ATV/height^2^ in early infancy. The ratio IA/SCA ATV remains significantly correlated with SCA in both the neonatal period and early infancy; the index IA/SCA^0.6^ is statistically optimal at both of these ages.

## Introduction

Early life factors are associated with adult adiposity,^1,2^ the metabolic syndrome^3^ and cardiovascular disease,^4,5^ and as a result, there is an increasing interest in early life adipose tissue (AT) development.^6,7^ When investigating factors affecting AT or analysing longitudinal changes, AT depots require normalisation for body size if comparisons between or within individuals are to be meaningful. For example, consider two infants immediately after birth, infant A weighs 3.3 kg while infant B weighs 4.2 kg. Both infant A and Infant B have identical total AT volumes (ATVs), 1.8 l, but different quantities of non-AT. For these infants, expressing ATVs as absolute values will conceal considerable differences in relative adiposity.

This is of particular importance during periods of rapid change in body size, such as infancy. To the best of our knowledge, optimal techniques for adjusting directly measured AT in early childhood have not been determined. Potential approaches include the use of multiple regression (including a measure of body size as a variable in a regression where AT is the outcome) and generation of an index for AT that is independent of body size. The index approach is advantageous in that it is easy to understand and compute and can be easily compared across different studies and populations. It is presumably for these reasons that indices are commonly used for adjustment.

There are three rationales for using an index to adjust body composition; these are to remove the effect of a denominator (usually a measure of body size), to correlate maximally with a specific outcome (such as insulin resistance) or to correlate maximally with risk (such as mortality).^8^ In pediatric practice, adjustment is commonly performed to address growth variability with the intention of minimising the correlation between the index and a measure of body size (the denominator). For two-component measures of body composition, this is most commonly performed by calculating percentage fat mass (FM), where body weight is the denominator. This approach is statistically flawed and conceptually problematic; the denominator, weight, is in part comprised of the numerator, AT, and is inverse correlated with non-adipose weight. In addition, adjustment for body weight underestimates adiposity in individuals with high adiposity, such as infants.^9, 10, 11^ Adjustment for height has been shown to have greater statistical validity for FM and fat-free mass (FFM) in pediatric populations.^10^ A further problem arises in that directly measured adiposity is quantified as volume rather than mass; conversion to AT mass introduces further inaccuracy through the assumptions required to convert volume into mass. Despite these problems, the measures percentage AT and percentage fat are still widely used.^6,7^

Another reason for adjustment of adiposity is to quantify metabolic load. In this situation, a measure of body composition is adjusted for another measure with which it is correlated but which has an opposing effect; for example FM, a component of metabolic risk, is often adjusted for FFM, a component that has an opposing metabolic effect as it incorporates organs and tissues maintaining homeostasis.^11,12^ Similarly, in adults internal abdominal (IA) AT, a component of metabolic risk^13^ is adjusted for subcutaneous abdominal (SCA) AT, a component in which accumulation is associated with beneficial metabolic effects,^14,15^ using the ratio IA/SCA. In an adult population, the AT ratio IA/SCA correlates more strongly with cardiometabolic risk than IA AT alone.^16^

Methods to adjust AT have not, to our knowledge, been defined for studies in infancy. The aims of this paper are to examine the statistical validity of commonly used indices and determine mathematically optimal approaches for adjusting ATV for body size, and for adjusting IA ATV relative to SCA ATV. An optimal index has minimal correlation with the denominator and allows comparison between different AT depots.

## Materials and Methods

### Data

Data from healthy infants born at term (37–42 gestational weeks), obtained during the course of research investigating adiposity in early life, were included in this investigation.^7,17, 18, 19^ Analyses were performed separately for the first month (0–30 days) and early infancy (42–91 days) because of the rapid change that occurs in adiposity in infancy.^20^

### Measurement techniques

For all infants, weight was measured using scales accurate to 0.2 g (Marsden Professional Baby Scale, London, UK) and length was measured with a Rollametre, accurate to 1 mm (Raven Equipment Ltd., Dunmow, Essex, UK). Anthropometric data are expressed as s.d. scores (SDS) relative to UK reference data.^21^ Total and regional ATV were measured in all infants using whole-body magnetic resonance imaging as previously described.^18,22^ Total ATV was calculated as the sum of six individually quantified AT compartments: superficial subcutaneous abdominal, superficial subcutaneous non-abdominal, deep subcutaneous abdominal, IA and internal non-abdominal as previously described^19^ (Supplementary Figure 1). To calculate the ratio SCA/IA, SCA ATV was calculated as the sum of superficial subcutaneous abdominal and deep subcutaneous abdominal AT compartments. For calculation of percentage fat, ATV was converted to FM using the formula:

FM mass, kg=ATV, litres × (density of AT: 0.987 kg l^−1^^(ref.23)^) × (proportion of AT comprised by fat at 1 month: 0.429^(ref.24)^).

### Calculation of validity

The validity of the following methods of adjusting total ATV for size was evaluated:

1. Percentage of fat (total FM (kg)/mass (kg) × 100)
2. Total ATV (litre)/length (m)
3. Using the mathematically optimal index for adjustment (also referred to as the Benn index^25^) in the form: AT index (ATI)=ATV (litres)/length(metres)^*p*^, using a mathematically optimal value of *p* (as defined below).

Correlation coefficients were calculated for each index with the measure of body size used in that index, to ascertain the degree to which these indices remained correlated with body size.

### Calculation of the optimal value for *p*

Determination of the optimal value of *p*, defined as minimal correlation between ATI and length, was performed using log-log regression;^10,25^ ATV and length were log-transformed using a natural log to the base e, log(ATV) was regressed against log(length) and the regression coefficient calculated. The optimal value for *p* is the regression coefficient.

### Individual AT depots

The above process was repeated for each individual depot at both points (the first month and early infancy). Calculating indices in this manner produced different values of *p* for each adjusted measure. Comparison between adjusted measures requires a single value of *p* suitable for use in all adjustments for size. A summary *p* at each time point, denoted *sp*, was therefore calculated from the median *p*, rounded to the nearest integer. The validity of *sp* was evaluated by calculating percentage variation in ATI^*sp*^ that was attributable to the denominator (length) for each AT depot as follows: the correlation between ATI^*sp*^ and length was calculated and the correlation coefficient *r*  was used to calculate the percentage of variation in ATI^*sp*^ that is attributable to length using the following equation:^10^


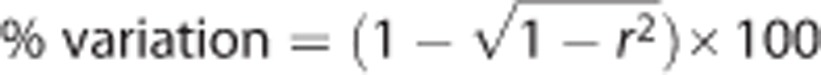


### Index for evaluation of metabolic load

To determine the degree to which IA/SCA is effective in minimising correlation with the denominator (SCA), the percentage variation in IA/SCA attributable to SCA was calculated as above. Determination of the statistically optimal value of *p* to which IA/SCA should be raised was calculated using log-log regression as described above.

## Results

Complete data (total and regional ATV and length) were available for 245 infants in the first month and 67 aged 42–91 days. Anthropometric data, ATV and commonly used ATIs are shown in Table 1; weight and length SDS show this population to be within 1 s.d. of the UK mean. Within the cohort, 16 infants were described by their parents as Asian, 8 as African, 9 as Afro-Caribbean, 174 as Caucasian and 30 as having mixed ethnicity (ethnicity was not recorded for 7 infants).

The indices percentage fat and AT/length remain significantly correlated with their denominators in the first month (*r*=0.33, *P*<0.001 and *r*=0.32, *P*<0.001 respectively; variation attributable to the denominator 5.6% and 5.3%, respectively). During the period 42–91 days, the correlation remained significant for percentage AT (*r*=0.38, *P*=0.001; variation attributable to the denominator 7.5%) but was not statistically significant for AT/length (*r*=0.23, *P*=0.06; variation attributable to the denominator 2.7%). Scatter plots illustrate the correlation between percentage FM and body mass (Supplementary Figure S2) and between AT/length and length (Supplementary Figure S3) both in the first month.

Regression analyses demonstrated that the gradient of the regression line relating log(ATV) to log(length) varies between different AT depots and between the two time periods (Table 2). The median regression coefficient was 3 in the neonatal period and 2 in early infancy; these values were used to calculate ATIs for each AT depot using the formula ATV/length^3^ for the first month and ATV/length^2^ for the period 42–91 days. Using this approach, the percentage variation in individual ATIs attributable to length was<2% for all AT depots at both ages except deep SCA in the neonatal period (3.7%) and IA early infancy (4%) (Table 3). These data indicate that the following indices, ATV/length^3^ in the first month and ATV/length^2^ for the period 42–91 days, are effectively uncorrelated with length and are suitable for adjustment of all AT depots.

**Figure 1:**
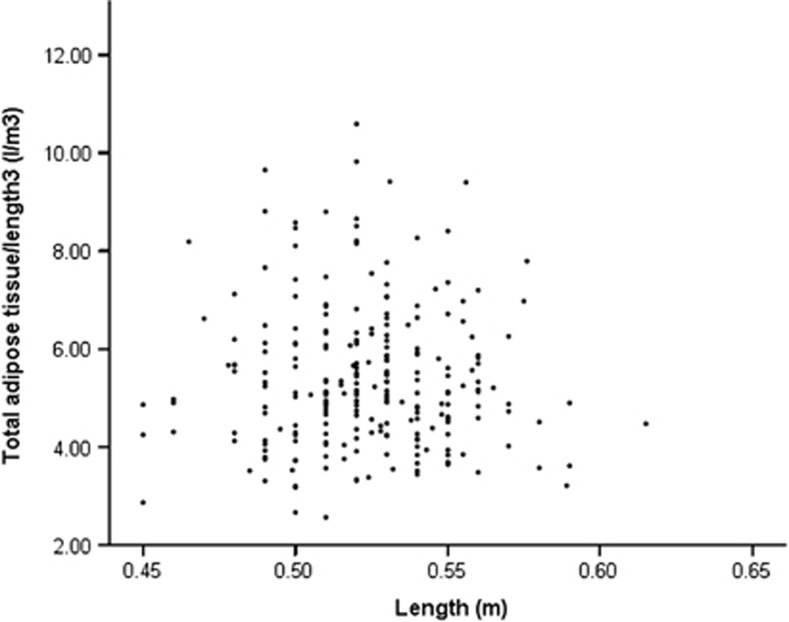
Scatter plot of correlation between total ATV/length^3^ and length in the first month; *r*=0.15, *P*=0.19 from Pearson correlation analysis.

In comparison with the other indices evaluated above (%fat, Supplementary Figure 2; total AT/length, Supplementary Figure 3), the correlation coefficient (*r*) between ATV/length^3^ and the length in the neonatal period is 0.15, *P*=0.19, Figure 1.

**Figure 2:**
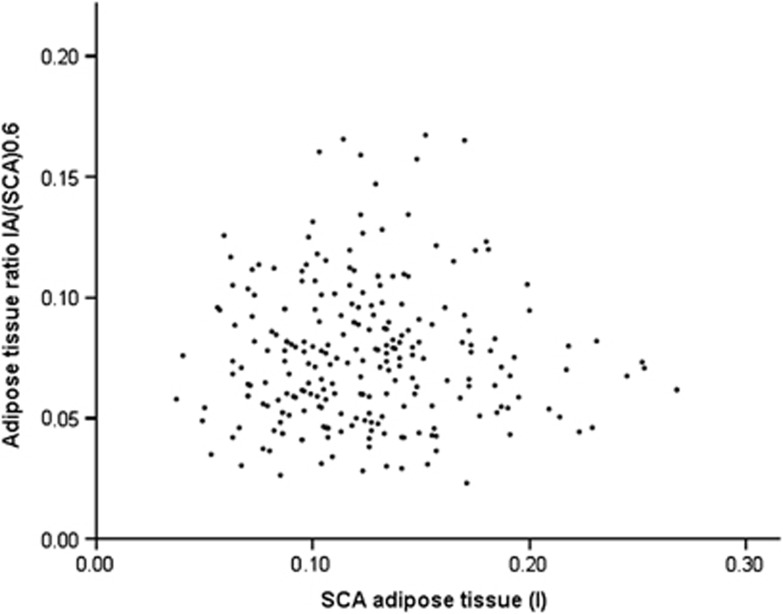
Scatter plot of correlation between IA/SCA^0.6^ AT and SCA AT in the first month; *r*=0.002, *P*=0.97 from Pearson correlation analysis.

Values for IA/SCA in the first month and in early infancy are given in Table 1. This index is significantly correlated with the denominator, SCA, at both points (first month *r*=−0.33, *P*<0.001, Supplementary Figure S4; 42–91 days *r*=−0.29, *P*=0.01). In the first month, there is an outlier value with a SCA of 0.37; repeat analysis after removing this outlier still demonstrates a significant association (*r*=−0.32, *P*<0.001). Log-log regression demonstrates that the optimal value of *p* for the index IA/SCA^*p*^ is 0.6 (*P*<0.001, 95% CI 0.5, 0.8) in the first month (Figure 2) and 0.6 (*P*<0.001, 95% CI 0.4, 0.9) for the period 42–91 days. Use of the nearest integer, 1 (IA/SCA), results in a percentage variation attributable to SCA of 5.6% in the first month and 4.3% in early infancy.

## Discussion

The advent of direct measurement techniques such as magnetic resonance imaging allows detailed quantification of adiposity and delineation of regional AT distribution; where body size is highly variable or rapidly changing, such as in infancy and childhood, meaningful comparisons require adjustment. Ratios or indices are widely used to adjust body composition, are easily interpreted and have been statistically validated in adults^26^ and children.^10^ Indices adjusting regional AT compartments have not been subject to similar investigation and are further complicated in that an index that effectively adjusts one AT compartment (by minimising the correlation with body size) may not be effective for another compartment (remaining highly correlated with body size). Ideally a single index would be applicable for use across all compartments and throughout infancy.

We show that in infancy adjustment of AT for body size using percentage FM or ATV/length is statistically problematic in that the index remains correlated with the denominator. Although the degree of variation attributable to body size in these two indices may not be considered excessive (up to 7.5%), important inaccuracies may result when comparing groups that contrast significantly in size (for example, comparing preterm with term infants). These problems can be minimised through the use of more statistically appropriate methods of adjustment.

We demonstrate that the appropriate power to which length should be raised to minimise correlation between ATV and size in infancy changes over time, differs between compartments and is as high as 4.8. Use of the values 3 in the first month (ATV/length^3^) and 2 in later infancy over the period 42–91 days (ATV/length^2^) results in indices that are mathematically valid, in that length explains<2% of the variance in the index for almost all AT compartments, while allowing meaningful comparison between different compartments. We propose that future infant studies in which comparison of regional AT compartments are required should adjust measures for body size using these indices. The neonatal period is one of the few instances where BMI remains correlated with length, hence use of the Ponderal index (which adjusts weight using length^3^) is appropriate.^27^ Data presented here demonstrate that in the first month AT and weight scale to length^3^, while later in infancy, and in keeping with adult studies,^28^ AT and weight^27^ scale to length^2^. A drawback to using ATV/length^3^ in the first month and ATV/length^2^ in early infancy is that this limits temporal comparisons. There are two common reasons for comparing adjusted adiposity over time, and we suggest that the optimal method to account for the change in adjustment indices between the neonatal and infant periods depends on the underlying rationale. One reason for comparison is to examine whether an adipose baby is more likely to become an adipose adult; in this case we suggest converting each outcome (ATV/length^3^ in the first month and ATV/length^2^ for later periods) into age- and sex-specific SDS in order to calculate their correlation coefficient. A second reason would be to plot a life course trend in adjusted adiposity; in this case, the adiposity outcome (ATV/length^3^ in the first month and ATV/length^2^ for later periods) SDS could be calculated and plotted on a common axis, or alternatively a pragmatic decision could be made to use ATV/length^2^ across the entire life course, accepting that a correlation between the adjusted measure and length would remain in the first month.

Molecular and metabolic differences between the deep and superficial abdominal subcutaneous AT depots are increasingly recognised,^29^ although these have not, to our knowledge, been incorporated into a ratio describing the metabolic load of abdominal adiposity. The ratio IA/SCA is, however, widely used for this purpose in adults^16,30^ and has been demonstrated to be a more useful marker of cardiometabolic outcomes than IA AT alone. It is for this reason that we have examined the ratio IA/SCA in infancy. Similar physiological validation between the ratio IA/SCA and insulin or glucose metabolism has not, to our knowledge, been performed in infancy. Here we demonstrate that this ratio is not statistically optimal (in that it remains significantly correlated with SCA) in healthy infants up to 3 months of age. Where observations are confined to early infancy, we suggest use of the index IA/SCA^0.6^. That the optimal index in infancy (IA/SCA^0.6^) differs from the one used in later childhood^31^ and adult life (IA/SCA) presents problems similar to those outlined above when temporal comparison is required. This can again be satisfactorily resolved by calculating and comparing age- and sex-specific SDS for the adiposity outcome (IA/SCA^0.6^ in infancy and IA/SCA in childhood).

The data we have used to explore indices and relationships between AT depots are from the largest cohort known to us of infants imaged using magnetic resonance imaging. A further strength is the inclusion of only healthy term infants, which allows the construction of reference indices. Limitations include the lack of physiological measures and hence our inability to relate the indices described with metabolism and the limited number of infants outside the neonatal period. The latter limitation is reflected in the wider confidence limits for analyses undertaken at the later time point. Differences in infant AT distribution in relation to ethnicity have been previously described;^19^ we were unable to examine whether the statistical validity of ATIs is influenced by ethnicity due to the predominantly Caucasian nature of this cohort.

In conclusion, current approaches, principally the use of percentage fat or percentage AT mass to adjust for infant size, have statistical limitations. We suggest that adjustment of AT depots for body size is most appropriate when done using the index ATV/length^3^ in the first month and the index ATV/length^2^ in later infancy up to 3 months of age. When a measure of the metabolic load of IA AT is required, we suggest use of the ratio IA/SCA^0.6^.

## Figures and Tables

**Table 1:** Demographic and adiposity data in the first month and for 42–91 days

	*First month*	*42–91 days*
Number	245	67
Scan age (days)	11 (4–16)	63 (58–71)
Weight (kg)	3.455 (0.563)	5.269 (0.752)
Weight SDS	−0.41 (0.91)	0.05 (0.93)
Length (m)	0.523 (0.027)	0.587 (0.030)
Length SDS	0.38 (1.12)	0.68 (1.50)
Total AT (l)	0.748 (0.627–0.907)	1.526 (1.332–1.798)
Superficial subcutaneous abdominal AT(l)	0.106 (0.082–0.127)	0.256 (0.213–0.321)
Superficial subcutaneous non-abdominal AT(l)	0.529 (0.449–0.656)	1.085 (0.934–1.270)
Deep subcutaneous abdominal AT(l)	0.015 (0.010–0.020)	0.041 (0.031–0.050)
Deep subcutaneous non-abdominal AT(l)	0.012 (0.009–0.015)	0.020 (0.016–0.023)
Internal abdominal AT(l)	0.020 (0.014–0.027)	0.030 (0.023–0.042)
Internal non-abdominal AT(l)	0.055 (0.045–0.070)	0.086 (0.071–0.118)
IA/SCA	0.17 (0.12–0.22)	0.10 (0.08–0.14)
Percentage fat	9.2 (8.0–10.5)	12.3 (10.8–14.0)
Total AT/length (l/m)	1.42 (1.19–1.70)	2.62 (2.22–3.10)

Abbreviations: AT, adipose tissue; IA, internal abdominal; SCA, subcutaneous abdominal; SDS, s.d. score. Data are median (interquartile range), except for weight and length where data are mean (s.d.).

**Table 2:** Regression coefficients, *β* (95% CI), from log-log regression analyses of adipose tissue compartments and length

*Adipose tissue*	*First month*		*42–91 days*	
	β	P	β	P
Total	3.0 (2.3, 3.6)	<0.001	2.1 (0.8, 3.4)	0.001
Superficial subcutaneous abdominal	3.1 (2.3, 3.9)	<0.001	2.0 (0.3, 3.7)	0.02
Superficial subcutaneous non-abdominal	3.1 (2.4, 3.7)	<0.001	2.1 (0.9, 3.4)	0.001
Deep subcutaneous abdominal	4.8 (3.6, 6.0)	<0.001	0.9 (-2.3, 4.1)	0.56
Deep subcutaneous non-abdominal	2.6 (1.7, 3.4)	<0.001	2.1 (-0.1, 4.4)	0.06
Internal abdominal	1.6 (0.6, 2.7)	0.003	−0.9 (-3.0, 1.3)	0.42
Internal non-abdominal	2.0 (1.2, 2.9)	<0.001	3.0 (1.3, 4.6)	0.001

Abbreviation: CI, confidence interval. Values are calculated by log transforming each adipose tissue compartment to the base e and regressing the log of the adipose tissue compartment on the log of the length. Median values for *β* in the first month and in the period 42–91 days are 3 and 2.1, respectively.

**Table 3:** Correlation coefficients (*r*) and *P* values (*P*) and percentage variation in adipose tissue indexes (ATI^3^ in the first month; ATI^2^ in the period 42–91 days) attributable to the denominator (length)

*Adipose tissue*	*First month*			*42–91 days*		
	r	P	*% variation*	r	P	*% variation*
Total	0.15	0.19	1.1	0.03	0.80	0.0
Superficial subcutaneous abdominal	0.17	0.14	1.5	0.02	0.88	0.0
Superficial subcutaneous non-abdominal	0.17	0.15	1.5	0.04	0.76	0.1
Deep subcutaneous abdominal	0.27	0.02	3.7	−0.04	0.74	0.1
Deep subcutaneous non-abdominal	0.01	0.90	0.0	0.08	0.51	0.3
Internal abdominal	−0.11	0.34	0.6	−0.28	0.02	4.0
Internal non-abdominal	−0.05	0.68	0.1	0.13	0.28	0.9

Abbreviation: ATI, adipose tissue index.

## Notes

CG has received support from Pfizer Nutrition to attend an educational conference; he declares no other conflict of interest. In the past 5 years, NM has received consultancy fees from Ferring Pharmaceuticals, speaker honorarium for an educational meeting funded by Nestle International in which they had no organisational involvement and grants from the Medical Research Council, National Institute of Heath Research, Westminster Children's Trust Fund, Child Growth Foundation, Action Medical Research, HCA International, Danone, Bliss, British Heart Foundation and Department of Health. Salary support for CG provided by Chelsea and Westminster Hospital Charity. The other authors declare no conflict of interest.
