# Supplementary material for: Adjustment of directly measured adipose tissue volume in infants
Source: Int J Obes (Lond). 2014 Apr 29;38(7):995–9. doi: 10.1038/ijo.2014.48 (PMC4088334; doi:10.1038/ijo.2014.48)
Supplement: Supplementary Figure 1 [file ijo201448x1.doc]

**Total**

Superficial subcutaneous

Deep subcutaneous

Internal

Abdominal superficial subcutaneous

Non-abdominal superficial subcutaneous

Abdominal deep subcutaneous

Non-abdominal deep subcutaneous

Internal abdominal

Internal non-abdominal

**Supplemental figure 1: Schematic representation of adipose tissue depots.** The adipose tissue depot subcutaneous abdominal (SCA) is comprised by the two greyed boxes (abdominal superficial subcutaneous and abdominal deep subcutaneous).
