# Supplementary material for: Adjustment of directly measured adipose tissue volume in infants
Source: Int J Obes (Lond). 2014 Apr 29;38(7):995–9. doi: 10.1038/ijo.2014.48 (PMC4088334; doi:10.1038/ijo.2014.48)
Supplement: Supplementary Figure 3 [file ijo201448x3.doc]

**
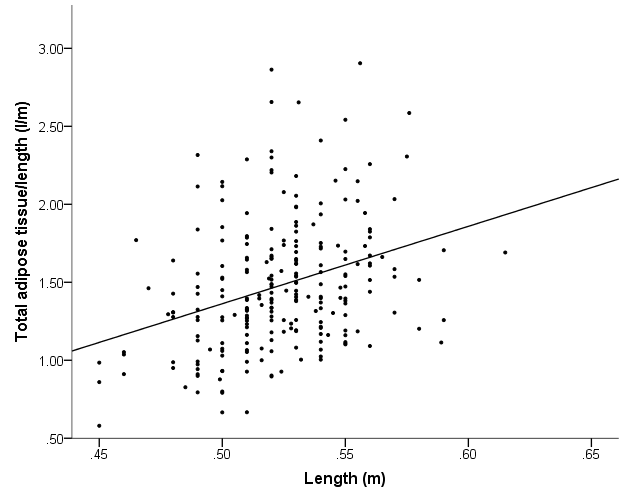
Supplemental figure 3: Scatter plot of correlation between (total adipose tissue volume/length) and length in the first month**; r=0.32, p<0.001 from Pearson correlation analysis.
