# Supplementary material for: Adjustment of directly measured adipose tissue volume in infants
Source: Int J Obes (Lond). 2014 Apr 29;38(7):995–9. doi: 10.1038/ijo.2014.48 (PMC4088334; doi:10.1038/ijo.2014.48)
Supplement: Supplementary Figure 4 [file ijo201448x4.doc]

**
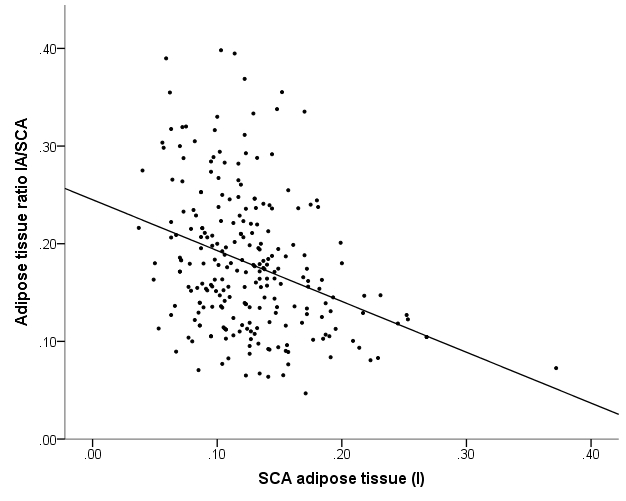
Supplemental figure 4: Scatter plot of correlation between IA/SCA adipose tissue and SCA adipose tissue in the first month**; r=-0.33, p<0.001 from Pearson correlation analysis.
